# Supplementary figures and images for: Genome-wide identification and expression analysis of SBP-box gene family reveal their involvement in hormone response and abiotic stresses in Chrysanthemum nankingense
Source: PeerJ. 2022 Oct 27;10:e14241. doi: 10.7717/peerj.14241 (PMC9618261; doi:10.7717/peerj.14241)

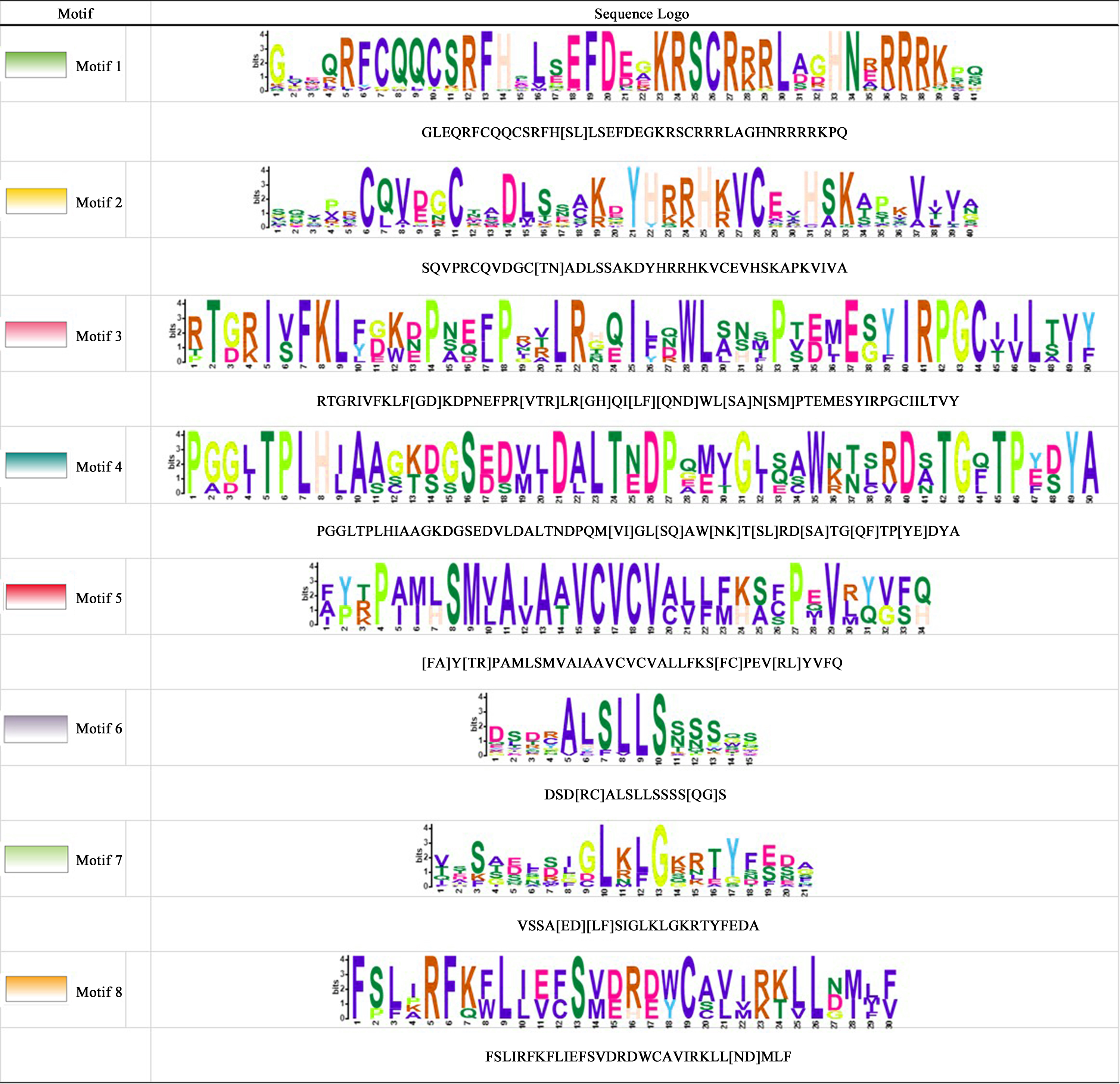

Supplement: Supplemental Information 11 — Conserved motifs showed by TBtools and sequence visualized by Weblogo and MEME websites. [file peerj-10-14241-s011.jpg]

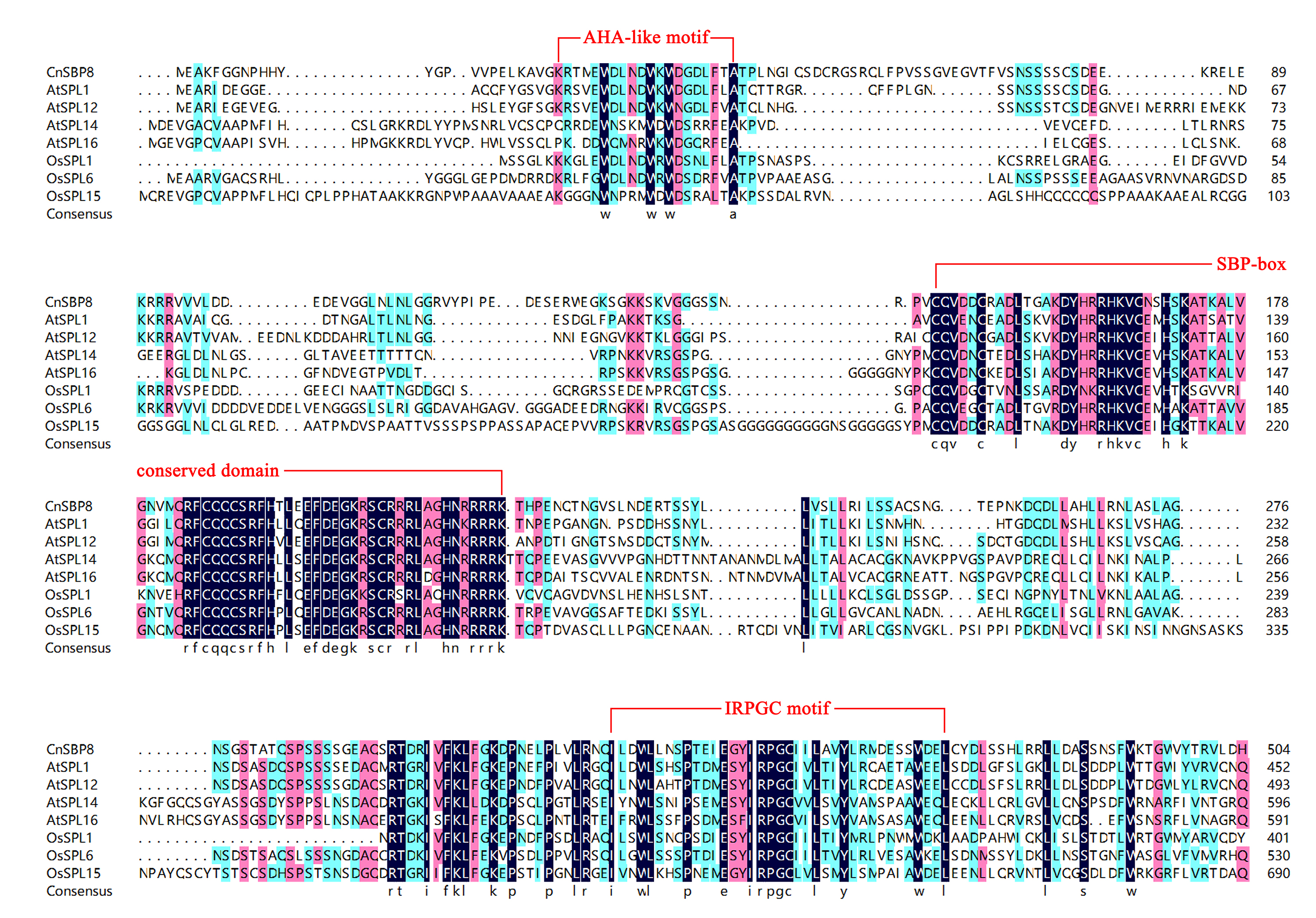

Supplement: Supplemental Information 12 — The location of the first red box upstream of SBP domain was AHA-like motif and the third of red box downstream of SBP domain was IRPGC motif with specific conserved aa residues. [file peerj-10-14241-s012.jpg]

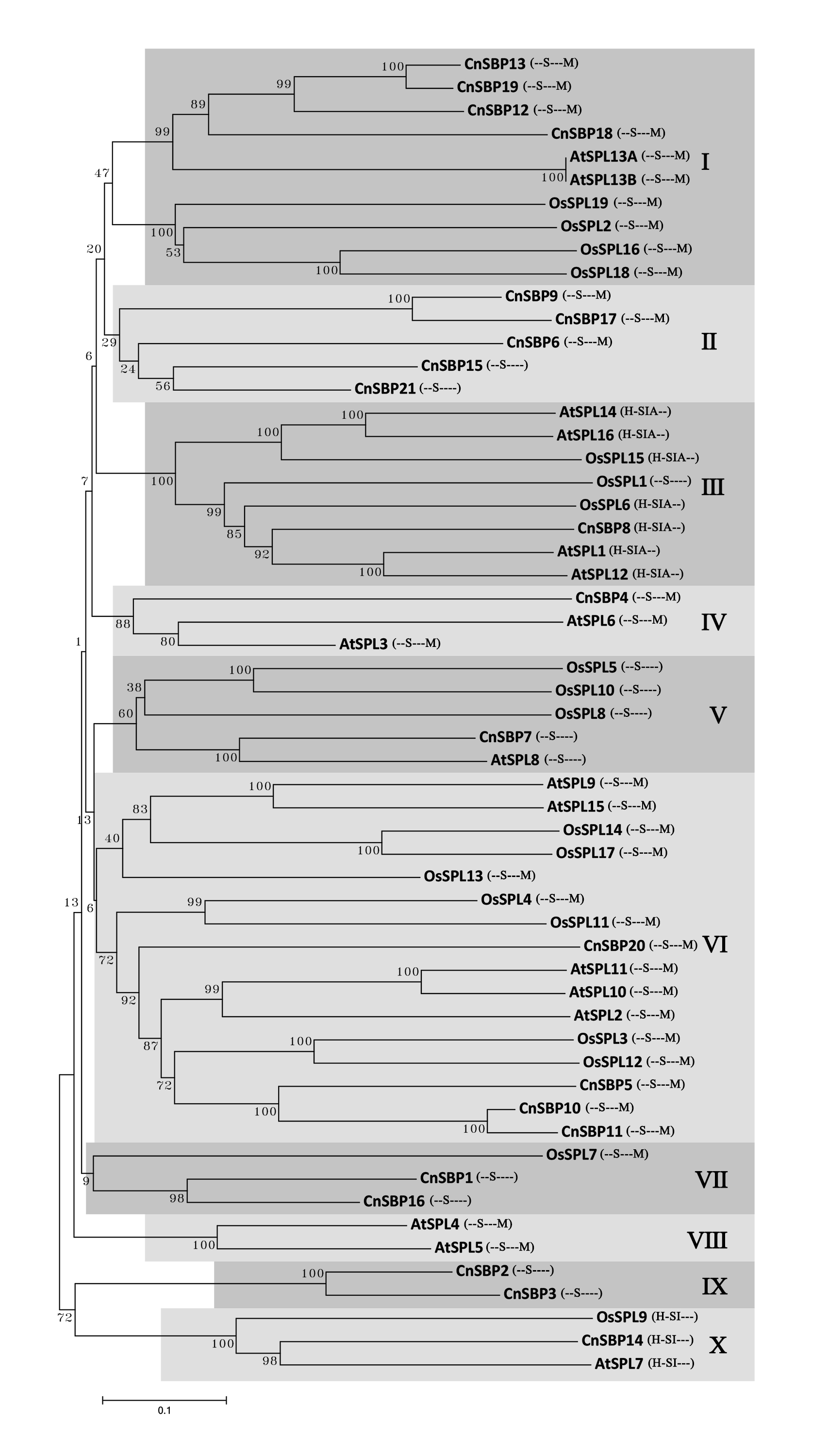

Supplement: Supplemental Information 13 — The conserved sequences characteristic for the different subfamilies summarized by a letter code between brackets at the end of gene names. H, AHA-like motif; S, SBP-domain; I, IRPGC-domain; A, ankyrin repeat region and M, MRE-element. [file peerj-10-14241-s013.jpg]

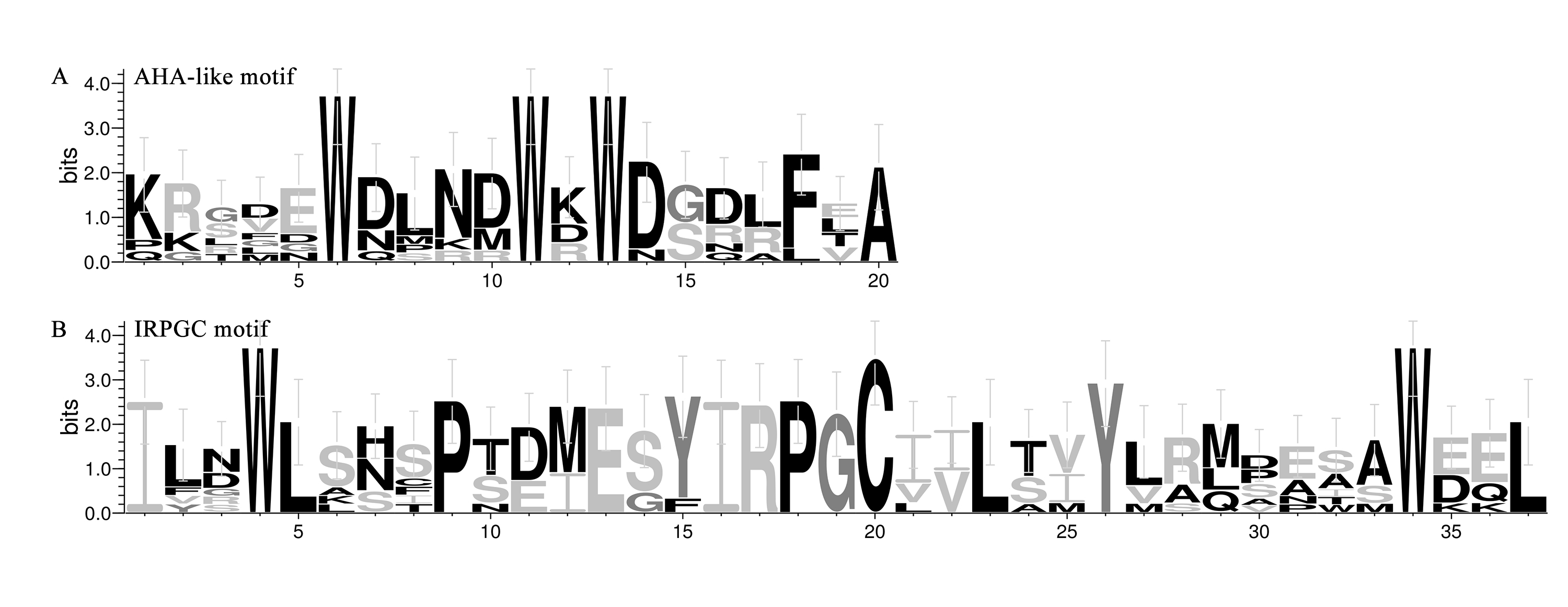

Supplement: Supplemental Information 14 — (A) Amino acid sequence logo of the AHA-like1 motif in eight CnSBPs. (B) Amino acid sequence logo of the IRPGC motif in eight CnSBPs. Each logo consisted of stacks of symbols, one stack for each position in the sequence. [file peerj-10-14241-s014.jpg]
