# Supplementary material for: Genome-wide identification and expression analysis of SBP-box gene family reveal their involvement in hormone response and abiotic stresses in Chrysanthemum nankingense
Source: PeerJ. 2022 Oct 27;10:e14241. doi: 10.7717/peerj.14241 (PMC9618261; doi:10.7717/peerj.14241)
Supplement: Supplemental Information 15 [file peerj-10-14241-s015.docx]

| Gene name | Primer Sequence | start | end |
| --- | --- | --- | --- |
| *CnSBP1* | Forward: 5-GGTGTTGGAGGAGGTTCGTC-3 | 85 | 104 |
|  | Reverse: 5-AACGACAACAGCTTGAGCCT-3 | 276 | 257 |
| *CnSBP2* | Forward: 5-AGGGGTTTAGCACGACACAA-3 | 291 | 310 |
|  | Reverse: 5-ACACCAAACAAGCATAGCCG-3 | 476 | 457 |
| *CnSBP3* | Forward: 5-GTATGTTCCCCTGCCGACAA-3 | 649 | 668 |
|  | Reverse: 5-CAAACCGGCATCTCAGTCCA-3 | 749 | 730 |
| *CnSBP4* | Forward: 5-CACCGCTTTTGCTACTCCCT-3 | 1203 | 1222 |
|  | Reverse: 5-AATATCCCGGTGCGAGTTCC-3 | 1342 | 1323 |
| *CnSBP5* | Forward: 5-CCTTGCTATGTTCGGGTCCA-3 | 33 | 52 |
|  | Reverse: 5-CCACCAACAACGCCTGAATG-3 | 152 | 133 |
| *CnSBP6* | Forward: 5-CATAACCGTCGCCGTAGGAA-3 | 295 | 314 |
|  | Reverse: 5-CCCACCATGCTACTCAAGGG-3 | 476 | 457 |
| *CnSBP7* | Forward: 5- CGCCAACTCTTGAAAACCCG-3 | 377 | 396 |
|  | Reverse: 5-ACTCACTAAAGCAGCGTCCA-3 | 573 | 554 |
| *CnSBP8* | Forward: 5-ACGCCACCCTCAAAAGTCAA-3 | 1137 | 1156 |
|  | Reverse: 5-GAGCACGAGAAGGTAAGGCA-3 | 1790 | 1771 |
| *CnSBP9* | Forward: 5-ATTCGTATGCCCGTGTGAGG-3 | 476 | 495 |
|  | Reverse: 5-GCTGATGCACTTGTGTGCTG-3 | 587 | 568 |
| *CnSBP10* | Forward: 5-GGTGCCAGAGCCGAGATATT-3 | 484 | 503 |
|  | Reverse: 5-TGATGAGGCATGGTGGGAAC-3 | 641 | 622 |
| *CnSBP11* | Forward: 5-ATTCGTGGGGTTCATCCGAG-3 | 926 | 945 |
|  | Reverse: 5-TGACTGTTGGTTAGCCTGCC-3 | 1062 | 1043 |
| *CnSBP12* | Forward: 5-TGGGTTCAATCATCCGGTGG-3 | 384 | 403 |
|  | Reverse: 5-TAGGGGTTGTTCCACATCGC-3 | 486 | 467 |
| *CnSBP13* | Forward: 5-GTTGCAGTTTGGTGGTCAGC-3 | 315 | 334 |
|  | Reverse: 5-GTGAGCGTAGTCCGAATGGT-3 | 414 | 395 |
| *CnSBP14* | Forward: 5-AGCTCCACTCATGCCCAAAC-3 | 709 | 728 |
|  | Reverse: 5-TGAGATCCGACCAGTTGTGC-3 | 870 | 851 |
| *CnSBP15* | Forward: 5-CGTTTACTCAAGACGGGGCA-3 | 53 | 72 |
|  | Reverse: 5-ATTCCGGTGGGTTACTTCCC-3 | 222 | 203 |
| *CnSBP16* | Forward: 5-ACTGCGTCCTCCAACCAAAA-3 | 10 | 29 |
|  | Reverse: 5-TTGTGTTGCCAGACGATCCA-3 | 160 | 141 |
| *CnSBP17* | Forward: 5-ATTCGTATGCCCGTGTGAGG-3 | 629 | 648 |
|  | Reverse: 5-GCTGATGCACTTGTGTGCTG-3 | 740 | 721 |
| *CnSBP18* | Forward: 5-CCCTATCCCGACCCACAAAC-3 | 663 | 682 |
|  | Reverse: 5-GGTGTGAGCCTGAACCTGAA-3 | 784 | 765 |
| *CnSBP19* | Forward: 5-AGGAATCGTCTTGATGGGCA-3 | 241 | 260 |
|  | Reverse: 5-CCTGGAGGGTGATTGAACCG-3 | 386 | 367 |
| *CnSBP20* | Forward: 5-AGACGGCTACAACATCCGAG-3 | 356 | 375 |
|  | Reverse: 5-CTACTTGACAGCGTGGGGAC-3 | 455 | 436 |
| *CnSBP21* | Forward: 5-GATGCAAGAGTGGGAACAGGA-3 | 63 | 83 |
|  | Reverse: 5-GTGTCTGTCCTCCGCTACTC-3 | 163 | 144 |

**Table S1 QRT-PCR primers list.**
