# Supplementary material for: Genome-wide identification and expression analysis of SBP-box gene family reveal their involvement in hormone response and abiotic stresses in Chrysanthemum nankingense
Source: PeerJ. 2022 Oct 27;10:e14241. doi: 10.7717/peerj.14241 (PMC9618261; doi:10.7717/peerj.14241)
Supplement: Supplemental Information 16 — Blue presents alpha helix; Green presents beta turn; Red presents extended strand; Pink presents random coil. [file peerj-10-14241-s016.docx]

**Table S2 The secondary and tertiary structures of *CnSBP* gene family.** Blue presents alpha helix; Green presents beta turn; Red presents extended strand; Pink presents random coil.

| Protein | Alpha helix (%) | Beta turn (%) | Random coli (%) | Extended strand (%) | Distribution of secondary structure elements | Tertiary structure prediction |  |
| --- | --- | --- | --- | --- | --- | --- | --- |
| CnSBP1 | 34.71 | 4.71 | 46.47 | 14.12 | 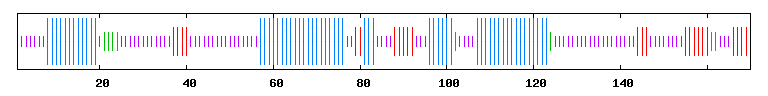 | 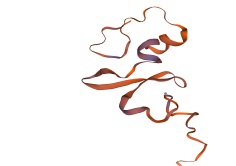 | |
| CnSBP2 | 21.47 | 9.2 | 44.17 | 25.15 | 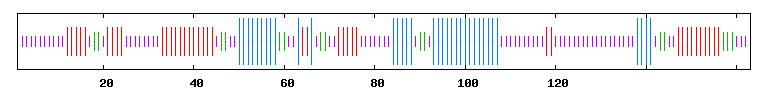 | 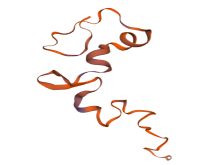 |  |
| CnSBP3 | 32.95 | 7.36 | 33.33 | 26.36 | 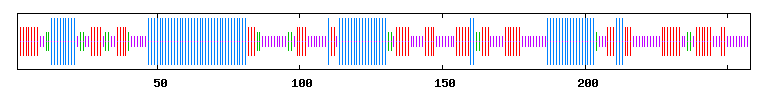 | 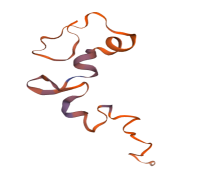 |  |
| CnSBP4 | 26.81 | 3.83 | 56.25 | 13.1 | 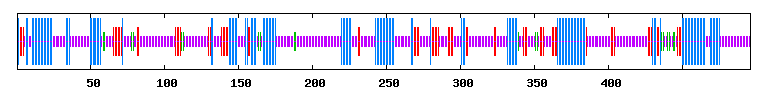 | \| 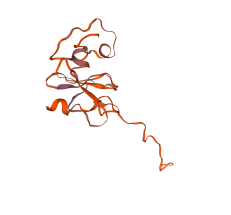 \| \| --- \| |  |
| CnSBP5 | 14.9 | 3.37 | 69.23 | 12.5 | 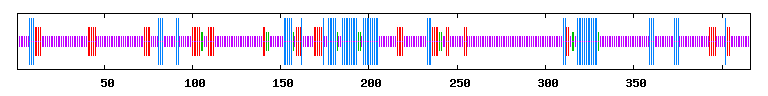 | 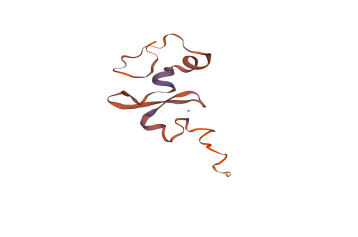 |  |
| CnSBP6 | 20.6 | 4.65 | 59.47 | 15.28 | 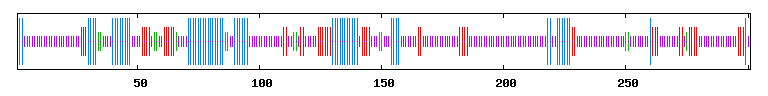 | 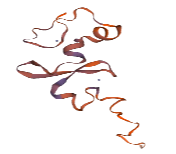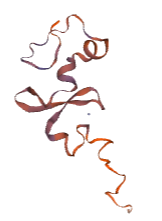 |  |
| CnSBP7 | 19.21 | 6.62 | 58.94 | 15.23 | 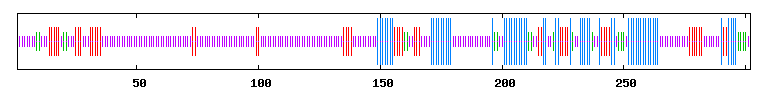 |  |  |
| CnSBP8 | 35.85 | 4.40 | 47.90 | 11.84 | 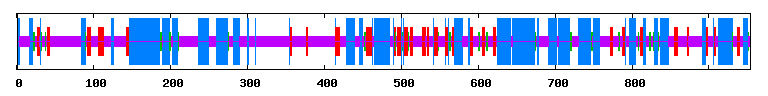 | 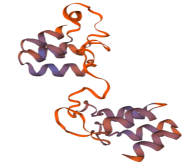 |  |
| CnSBP9 | 17.87 | 3.09 | 67.35 | 11.68 | 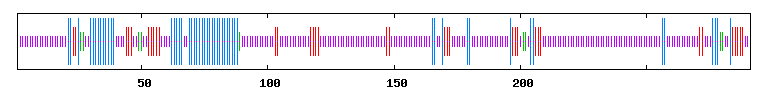 | 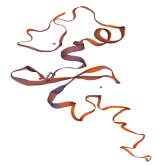 |  |
| CnSBP10 | 24.91 | 3.97 | 60.65 | 10.47 | 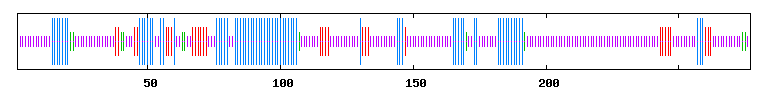 | 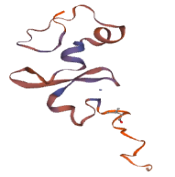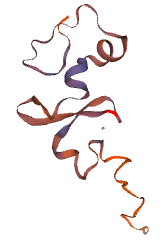 |  |
| CnSBP11 | 19.08 | 5.34 | 58.78 | 16.79 | 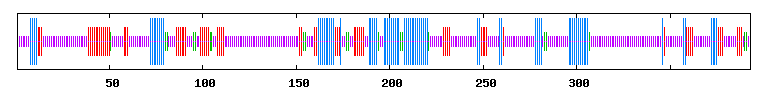 |  |  |
| CnSBP12 | 23.18 | 6.01 | 50.21 | 20.60 | 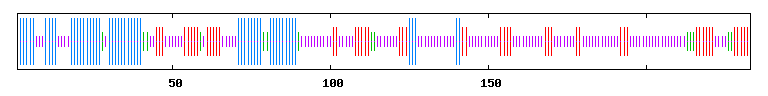 | 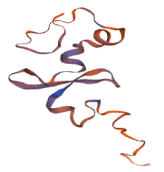 |  |
| CnSBP13 | 25.89 | 5.08 | 56.85 | 12.18 | 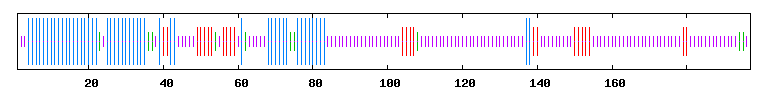 | 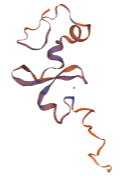 |  |
| CnSBP14 | 35.00 | 5.11 | 39.67 | 20.22 | 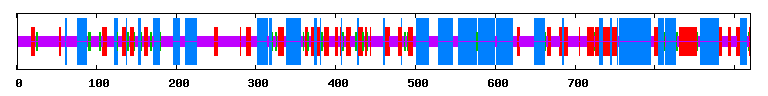 | \| 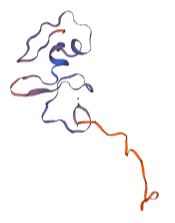 \| \| --- \| |  |
| CnSBP15 | 44.29 | 5.71 | 41.90 | 8.10 | 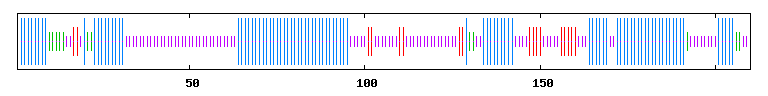 | 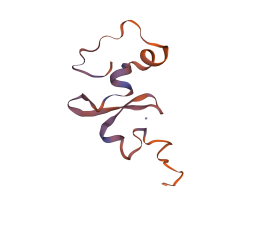 |  |
| CnSBP16 | 38.51 | 6.76 | 42.57 | 12.16 | 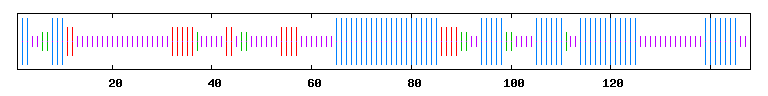 | 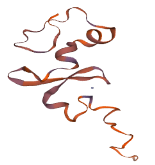 |  |
| CnSBP17 | 25.81 | 2.90 | 51.94 | 19.35 | 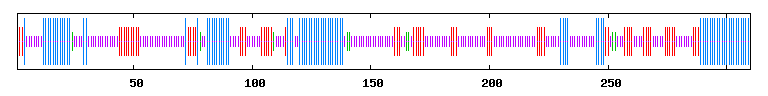 | 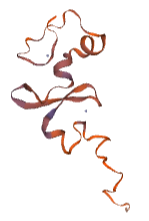 |  |
| CnSBP18 | 16.46 | 3.29 | 64.56 | 15.70 | 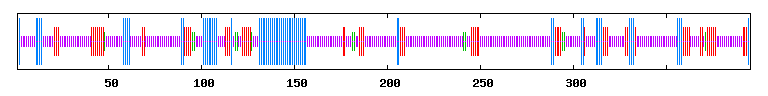 | 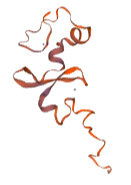 |  |
| CnSBP19 | 23.35 | 7.61 | 56.85 | 12.18 | 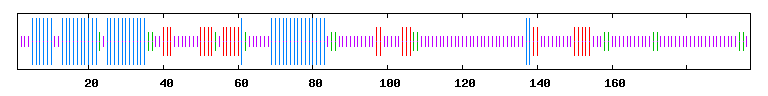 | 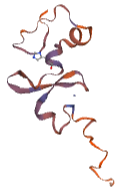 |  |
| CnSBP20 | 19.63 | 4.44 | 60.05 | 15.89 | 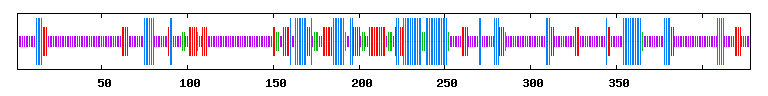 | 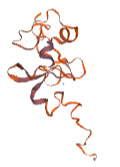 |  |
| CnSBP21 | 40.85 | 4.23 | 45.77 | 9.15 | 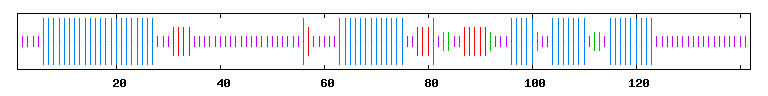 | 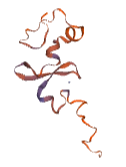 |  |
