# Supplementary material for: Genome-wide identification and expression analysis of SBP-box gene family reveal their involvement in hormone response and abiotic stresses in Chrysanthemum nankingense
Source: PeerJ. 2022 Oct 27;10:e14241. doi: 10.7717/peerj.14241 (PMC9618261; doi:10.7717/peerj.14241)
Supplement: Supplemental Information 17 [file peerj-10-14241-s017.docx]

**Table S3 Conserved domain sequences of CnSBP proteins.**

| Gene | Amino acids sequence |
| --- | --- |
| CnSBP1 | CQAEKCTSNLAEAKQYHRRHRVCESHAKAQAVVVAGIHQRFCQQCSRFHELSEFDEAKRSCRRRLAGHNERRRK |
| CnSBP2 | CQVDCCGDDLRVAKQYHQKHRVCELHTKAPLVLVGGLPRRFHEVSEFDDAKRSCRRGLARHNEVRRQ |
| CnSBP3 | CQVDCCGDDLRAAKQYHQKHRVCEVHTKAPVVLVGGLPRRFHEVSEFDDAKRSCRRGLARHNEVRRQ |
| CnSBP4 | CQVHGCSKSLVSCKDYHKRHKVCELHSKTAKVIVNGIVQRFCQQCSRFHLLSEFDDGKRSCRKRLADHNERRRK |
| CnSBP5 | CQVMGCDRDLSSAKDYHRKHRVCDVHSKSLKVIVAGLERRFCQQCSRFHGLPEFDGKKRSCRKRLADHNARRK |
| CnSBP6 | CLVDGCTADLGQCREYHRRHKVCEIHSKTPKVTIAGRDQRFCQQCSRFHSLAEFDEGKRSCRKRLDGHNRRRRK |
| CnSBP7 | CQAEGCNADLTHAKHYHRRHKVCEFHSKASTVITAGLTQRFCQQCSRFHLLSEFDNGKRSCRKRLADHNRRRRK |
| CnSBP8 | CQVDDCRADLTGAKDYHRRHKVCNSHSKATKALVGNVMQRFCQQCSRFHTLEEFDEGKRSCRRRLAGHNRRRRK |
| CnSBP9 | CLVDGCIADLSSCRDYHRRHRVCETHSKTPIVTIGGKDQRFCQQCSRFHPLGEFDEVKRSCRKRLDGHNRRRRK |
| CnSBP10 | CQVEGCNLDLSSSKEYHRKHKVCASHSKSPKVIVAGVERRFCQQCSRFHSMSEFDEEKRSCRRRLSDHNARRRK |
| CnSBP11 | CQVEGCNLDLSSSKEYHRKHKVCASHSKSPKVIVAGVERRFCQQCSRFHSMSEFDEEKRSCRRRLSDHNARRRK |
| CnSBP12 | CLVDGCMDDLRNCRMYHRRHKVCEAHSKSPYVLINGQTLRFCQQCSRFQSLEEFDEEKRSCRKRLAGHNRRRK |
| CnSBP13 | CLVDGCMDDLSNCRKYHQKHKICEAHSKSPQVLINGQTLRFCQQCSRFHSLEEFDEEKRSCRKRLDGHNRRRRK |
| CnSBP14 | CQVPGCEVDISELKGYHKRHRVCLRCANAGSVVLDGCDKRYCQQCGKFHVLSDFDEGKRSCRRKLERHNNRRRRK |
| CnSBP15 | CQVEGCTTDMTNCKTYHRRHKVCEVHAKAPIVVTNGCQQRFCQQCSRFHDLSEFDDAKRSCRRRLAGHNERRRK |
| CnSBP16 | CQADNCTSDLSEAKQYHRRHKVCELHAKAQAVIVAGTHQRFCQQCSRFHELSEFDDAKRSCRRRLAGHNERRRK |
| CnSBP17 | CLVDGCIADLSSCRDYHRRHRVCETHSKTPIVTIGGKDQRFCQQCSRFHPLGEFDEVKRSCRKRLDGHNRRRRK |
| CnSBP18 | CLVDGCNADLSGSKEYHRRHKVCEVHSKTALVLIHGQKQRFCQQCSRFHSLEEFDEGKRSCRKRLDGHNRRRRK |
| CnSBP19 | CLVDGCMDDLSNCRKYHQKHKVCEAHSKSPQVLINGQTLRFCQQCSRFHSLEEFDEEKRSCRNRLDGHNRRRRK |
| CnSBP20 | CQVEGCNLDLVSAKDYHRRHRICANHSKSPKVIVAGMERRFCQQCSRKVTFIKNNTHIGFLIHHLFLTSNKLTLRWVISRFHDLSEFDDRKRSCRRRLSAHNARRRR |
| CnSBP21 | CQVEECITDMSRCKTYHRRHKVCEIHAKAPIVVIGGRQQRFCQQCSRFHDLTEFDDAKRSCRRRLAGHNERRRK |
