# Supplementary material for: Genome-wide identification and expression analysis of SBP-box gene family reveal their involvement in hormone response and abiotic stresses in Chrysanthemum nankingense
Source: PeerJ. 2022 Oct 27;10:e14241. doi: 10.7717/peerj.14241 (PMC9618261; doi:10.7717/peerj.14241)
Supplement: Supplemental Information 18 [file peerj-10-14241-s018.docx]

**Table S4 Ka, Ks and Ka/Ks values calculated for homologous *SBP-box* gene pairs in *C. nankingense* and *Arabidopsis.***

| *Cn-Cn* | Ks | Ka | Ka/Ks | *Cn-At* | Ks | Ka | Ka/Ks |
| --- | --- | --- | --- | --- | --- | --- | --- |
| *CnSBP1/CnSBP16* | 0.1365 | 0.0876 | 0.64 | *CnSBP4/AtSPL6* | 0.9103 | 0.6450 | 0.71 |
| *CnSBP2/CnSBP3* | 0.1967 | 0.1498 | 0.76 | *CnSBP7/AtSPL8* | 0.5823 | 0.7007 | 1.20 |
| *CnSBP5/CnSBP10* | 0.5333 | 0.4377 | 0.82 | *CnSBP8/AtSPL1* | 0.726 | 0.5042 | 0.69 |
| *CnSBP5/CnSBP11* | 0.7387 | 0.3592 | 0.49 | *CnSBP8/AtSPL12* | 0.7276 | 0.5076 | 0.70 |
| *CnSBP9/CnSBP17* | 0.0169 | 0.0046 | 0.27 | *CnSBP14/AtSPL7* | 0.7365 | 0.5797 | 0.79 |
| *CnSBP10/CnSBP11* | 0.0103 | 0.0035 | 0.34 | *CnSBP18/AtSPL13* | 0.6348 | 0.5212 | 0.82 |
| *CnSBP12/CnSBP13* | 0.2475 | 0.1334 | 0.54 |  |  |  |  |
| *CnSBP12/CnSBP19* | 0.2611 | 0.1384 | 0.53 |  |  |  |  |
| *CnSBP13/CnSBP19* | 0.0609 | 0.0311 | 0.51 |  |  |  |  |
| *CnSBP15/CnSBP21* | 0.9279 | 0.2275 | 0.25 |  |  |  |  |
