# Supplementary material for: Genome-wide identification and expression analysis of SBP-box gene family reveal their involvement in hormone response and abiotic stresses in Chrysanthemum nankingense
Source: PeerJ. 2022 Oct 27;10:e14241. doi: 10.7717/peerj.14241 (PMC9618261; doi:10.7717/peerj.14241)
Supplement: Supplemental Information 19 [file peerj-10-14241-s019.docx]

| **Table S5 Critical *cis*-regulatory elements distribution of the *CnSBP* genes promoters.** | | |
| --- | --- | --- |
| Gene name | Annotation | |
| CnSBP1 | 3-AF1 binding site | light-responsiveness element |
| CnSBP1 | ACE | light-responsiveness element |
| CnSBP1 | GT1-motif | light-responsiveness element |
| CnSBP1 | GT1-motif | light-responsiveness element |
| CnSBP1 | GT1-motif | light-responsiveness element |
| CnSBP1 | G-Box | light-responsiveness element |
| CnSBP1 | G-Box | light-responsiveness element |
| CnSBP1 | G-Box | light-responsiveness element |
| CnSBP1 | G-Box | light-responsiveness element |
| CnSBP1 | G-Box | light-responsiveness element |
| CnSBP1 | G-Box | light-responsiveness element |
| CnSBP1 | MRE | light-responsiveness element |
| CnSBP1 | ABRE | auxin-responsive element |
| CnSBP1 | ABRE | auxin-responsive element |
| CnSBP1 | ABRE | auxin-responsive element |
| CnSBP1 | ABRE | auxin-responsive element |
| CnSBP1 | ABRE | auxin-responsive element |
| CnSBP1 | ABRE | auxin-responsive element |
| CnSBP1 | ARE | cis-acting regulatory element essential for the anaerobic induction |
| CnSBP1 | ARE | cis-acting regulatory element essential for the anaerobic induction |
| CnSBP1 | ARE | cis-acting regulatory element essential for the anaerobic induction |
| CnSBP1 | ARE | cis-acting regulatory element essential for the anaerobic induction |
| CnSBP1 | ARE | cis-acting regulatory element essential for the anaerobic induction |
| CnSBP1 | ARE | cis-acting regulatory element essential for the anaerobic induction |
| CnSBP1 | CGTCA-motif | cis-acting regulatory element involved in the MeJA-responsiveness |
| CnSBP1 | TGACG-motif | cis-acting regulatory element involved in the MeJA-responsiveness |
| CnSBP1 | LTR | cis-acting element involved in low-temperature responsiveness |
| CnSBP1 | MBS | MYB binding site involved in drought-inducibility |
| CnSBP1 | P-box | gibberellin-responsive element |
| CnSBP1 | RY-element | cis-acting regulatory element involved in seed-specific regulation |
| CnSBP1 | TGA-element | auxin-responsive element |
| CnSBP1 | circadian | cis-acting regulatory element involved in circadian control |
| CnSBP2 | GT1-motif | light-responsiveness element |
| CnSBP2 | G-Box | light-responsiveness element |
| CnSBP2 | G-Box | light-responsiveness element |
| CnSBP2 | G-Box | light-responsiveness element |
| CnSBP2 | ACE | light-responsiveness element |
| CnSBP2 | AE-box | light-responsiveness element |
| CnSBP2 | Box 4 | light-responsiveness element |
| CnSBP2 | Box 4 | light-responsiveness element |
| CnSBP2 | LAMP-element | light-responsiveness element |
| CnSBP2 | ABRE | auxin-responsive element |
| CnSBP2 | ABRE | auxin-responsive element |
| CnSBP2 | ABRE | auxin-responsive element |
| CnSBP2 | ARE | cis-acting regulatory element essential for the anaerobic induction |
| CnSBP2 | ARE | cis-acting regulatory element essential for the anaerobic induction |
| CnSBP2 | CGTCA-motif | cis-acting regulatory element involved in the MeJA-responsiveness |
| CnSBP2 | CGTCA-motif | cis-acting regulatory element involved in the MeJA-responsiveness |
| CnSBP2 | TGACG-motif | cis-acting regulatory element involved in the MeJA-responsiveness |
| CnSBP2 | TGACG-motif | cis-acting regulatory element involved in the MeJA-responsiveness |
| CnSBP2 | LTR | cis-acting element involved in low-temperature responsiveness |
| CnSBP2 | MBS | MYB binding site involved in drought-inducibility |
| CnSBP2 | O2-site | cis-acting regulatory element involved in zein metabolism regulation |
| CnSBP2 | O2-site | cis-acting regulatory element involved in zein metabolism regulation |
| CnSBP2 | TGA-element | auxin-responsive element |
| CnSBP3 | Sp1 | light-responsiveness element |
| CnSBP3 | LAMP-element | light-responsiveness element |
| CnSBP3 | I-box | light-responsiveness element |
| CnSBP3 | TCT-motif | light-responsiveness element |
| CnSBP3 | G-box | light-responsiveness element |
| CnSBP3 | ABRE | auxin-responsive element |
| CnSBP3 | ARE | cis-acting regulatory element essential for the anaerobic induction |
| CnSBP3 | ARE | cis-acting regulatory element essential for the anaerobic induction |
| CnSBP3 | ARE | cis-acting regulatory element essential for the anaerobic induction |
| CnSBP3 | CGTCA-motif | cis-acting regulatory element involved in the MeJA-responsiveness |
| CnSBP3 | TGACG-motif | cis-acting regulatory element involved in the MeJA-responsiveness |
| CnSBP3 | GARE-motif | gibberellin-responsive element |
| CnSBP3 | MBS | MYB binding site involved in drought-inducibility |
| CnSBP3 | O2-site | cis-acting regulatory element involved in zein metabolism regulation |
| CnSBP3 | O2-site | cis-acting regulatory element involved in zein metabolism regulation |
| CnSBP3 | RY-element | cis-acting regulatory element involved in seed-specific regulation |
| CnSBP3 | TATC-box | cis-acting element involved in gibberellin-responsiveness |
| CnSBP3 | TGA-element | auxin-responsive element |
| CnSBP4 | GATA-motif | light-responsiveness element |
| CnSBP4 | I-box | light-responsiveness element |
| CnSBP4 | G-box | light-responsiveness element |
| CnSBP4 | G-box | light-responsiveness element |
| CnSBP4 | TCT-motif | light-responsiveness element |
| CnSBP4 | ABRE | auxin-responsive element |
| CnSBP4 | ARE | cis-acting regulatory element essential for the anaerobic induction |
| CnSBP4 | ARE | cis-acting regulatory element essential for the anaerobic induction |
| CnSBP4 | HD-Zip | element involved in differentiation of the palisade mesophyll cells |
| CnSBP5 | GT1-motif | light-responsiveness element |
| CnSBP5 | GATA-motif | light-responsiveness element |
| CnSBP5 | TCT-motif | light-responsiveness element |
| CnSBP5 | ABRE | auxin-responsive element |
| CnSBP5 | ABRE | auxin-responsive element |
| CnSBP5 | ABRE | auxin-responsive element |
| CnSBP5 | ARE | cis-acting regulatory element essential for the anaerobic induction |
| CnSBP5 | ARE | cis-acting regulatory element essential for the anaerobic induction |
| CnSBP5 | ARE | cis-acting regulatory element essential for the anaerobic induction |
| CnSBP5 | ARE | cis-acting regulatory element essential for the anaerobic induction |
| CnSBP5 | HD-Zip | element involved in differentiation of the palisade mesophyll cells |
| CnSBP5 | LTR | cis-acting element involved in low-temperature responsiveness |
| CnSBP5 | TC-rich repeats | cis-acting element involved in defense and stress responsiveness |
| CnSBP5 | TC-rich repeats | cis-acting element involved in defense and stress responsiveness |
| CnSBP6 | I-box | light-responsiveness element |
| CnSBP6 | GT1-motif | light-responsiveness element |
| CnSBP6 | TCT-motif | light-responsiveness element |
| CnSBP6 | TCT-motif | light-responsiveness element |
| CnSBP6 | G-box | light-responsiveness element |
| CnSBP6 | ACE | light-responsiveness element |
| CnSBP6 | CGTCA-motif | cis-acting regulatory element involved in the MeJA-responsiveness |
| CnSBP6 | CGTCA-motif | cis-acting regulatory element involved in the MeJA-responsiveness |
| CnSBP6 | CGTCA-motif | cis-acting regulatory element involved in the MeJA-responsiveness |
| CnSBP6 | CGTCA-motif | cis-acting regulatory element involved in the MeJA-responsiveness |
| CnSBP6 | CGTCA-motif | cis-acting regulatory element involved in the MeJA-responsiveness |
| CnSBP6 | CGTCA-motif | cis-acting regulatory element involved in the MeJA-responsiveness |
| CnSBP6 | TGACG-motif | cis-acting regulatory element involved in the MeJA-responsiveness |
| CnSBP6 | TGACG-motif | cis-acting regulatory element involved in the MeJA-responsiveness |
| CnSBP6 | TGACG-motif | cis-acting regulatory element involved in the MeJA-responsiveness |
| CnSBP6 | TGACG-motif | cis-acting regulatory element involved in the MeJA-responsiveness |
| CnSBP6 | TGACG-motif | cis-acting regulatory element involved in the MeJA-responsiveness |
| CnSBP6 | TGACG-motif | cis-acting regulatory element involved in the MeJA-responsiveness |
| CnSBP6 | GARE-motif | gibberellin-responsive element |
| CnSBP6 | P-box | gibberellin-responsive element |
| CnSBP6 | RY-element | cis-acting regulatory element involved in seed-specific regulation |
| CnSBP6 | circadian | cis-acting regulatory element involved in circadian control |
| CnSBP7 | 3-AF1 binding site | light-responsiveness element |
| CnSBP7 | ACE | light-responsiveness element |
| CnSBP7 | Box 4 | light-responsiveness element |
| CnSBP7 | G-Box | light-responsiveness element |
| CnSBP7 | G-Box | light-responsiveness element |
| CnSBP7 | G-Box | light-responsiveness element |
| CnSBP7 | G-Box | light-responsiveness element |
| CnSBP7 | GT1-motif | light-responsiveness element |
| CnSBP7 | GT1-motif | light-responsiveness element |
| CnSBP7 | GT1-motif | light-responsiveness element |
| CnSBP7 | Sp1 | light-responsiveness element |
| CnSBP7 | Box 4 | light-responsiveness element |
| CnSBP7 | ABRE | auxin-responsive element |
| CnSBP7 | ABRE | auxin-responsive element |
| CnSBP7 | ABRE | auxin-responsive element |
| CnSBP7 | ABRE | auxin-responsive element |
| CnSBP7 | ABRE | auxin-responsive element |
| CnSBP7 | ABRE | auxin-responsive element |
| CnSBP7 | ARE | cis-acting regulatory element essential for the anaerobic induction |
| CnSBP7 | ARE | cis-acting regulatory element essential for the anaerobic induction |
| CnSBP7 | ARE | cis-acting regulatory element essential for the anaerobic induction |
| CnSBP7 | ARE | cis-acting regulatory element essential for the anaerobic induction |
| CnSBP7 | CGTCA-motif | cis-acting regulatory element involved in the MeJA-responsiveness |
| CnSBP7 | CGTCA-motif | cis-acting regulatory element involved in the MeJA-responsiveness |
| CnSBP7 | CGTCA-motif | cis-acting regulatory element involved in the MeJA-responsiveness |
| CnSBP7 | TGACG-motif | cis-acting regulatory element involved in the MeJA-responsiveness |
| CnSBP7 | TGACG-motif | cis-acting regulatory element involved in the MeJA-responsiveness |
| CnSBP7 | TGACG-motif | cis-acting regulatory element involved in the MeJA-responsiveness |
| CnSBP7 | GC-motif | enhancer-like element involved in anoxic specific inducibility |
| CnSBP7 | HD-Zip | element involved in differentiation of the palisade mesophyll cells |
| CnSBP7 | LTR | cis-acting element involved in low-temperature responsiveness |
| CnSBP7 | MSA-like | cis-acting element involved in cell cycle regulation |
| CnSBP7 | TCA-element | cis-acting element involved in salicylic acid responsiveness |
| CnSBP7 | TGA-element | auxin-responsive element |
| CnSBP8 | AAAC-motif | light-responsiveness element |
| CnSBP8 | G-Box | light-responsiveness element |
| CnSBP8 | G-Box | light-responsiveness element |
| CnSBP8 | GATA-motif | light-responsiveness element |
| CnSBP8 | chs-CMA1a | light-responsiveness element |
| CnSBP8 | ACE | light-responsiveness element |
| CnSBP8 | ACE | light-responsiveness element |
| CnSBP8 | ABRE | auxin-responsive element |
| CnSBP8 | ABRE | auxin-responsive element |
| CnSBP8 | ARE | cis-acting regulatory element essential for the anaerobic induction |
| CnSBP8 | ARE | cis-acting regulatory element essential for the anaerobic induction |
| CnSBP8 | ARE | cis-acting regulatory element essential for the anaerobic induction |
| CnSBP8 | ARE | cis-acting regulatory element essential for the anaerobic induction |
| CnSBP8 | O2-site | cis-acting regulatory element involved in zein metabolism regulation |
| CnSBP8 | P-box | gibberellin-responsive element |
| CnSBP8 | TC-rich repeats | cis-acting element involved in defense and stress responsiveness |
| CnSBP8 | TC-rich repeats | cis-acting element involved in defense and stress responsiveness |
| CnSBP8 | TC-rich repeats | cis-acting element involved in defense and stress responsiveness |
| CnSBP8 | TCA-element | cis-acting element involved in salicylic acid responsiveness |
| CnSBP8 | circadian | cis-acting regulatory element involved in circadian control |
| CnSBP9 | GT1-motif | light-responsiveness element |
| CnSBP9 | GATA-motif | light-responsiveness element |
| CnSBP9 | LAMP-element | light-responsiveness element |
| CnSBP9 | ARE | cis-acting regulatory element essential for the anaerobic induction |
| CnSBP9 | ARE | cis-acting regulatory element essential for the anaerobic induction |
| CnSBP9 | AuxRR-core | cis-acting regulatory element involved in auxin responsiveness |
| CnSBP9 | CGTCA-motif | cis-acting regulatory element involved in the MeJA-responsiveness |
| CnSBP9 | TGACG-motif | cis-acting regulatory element involved in the MeJA-responsiveness |
| CnSBP9 | MBS | MYB binding site involved in drought-inducibility |
| CnSBP9 | TATC-box | cis-acting element involved in gibberellin-responsiveness |
| CnSBP9 | TCA-element | cis-acting element involved in salicylic acid responsiveness |
| CnSBP9 | TCA-element | cis-acting element involved in salicylic acid responsiveness |
| CnSBP9 | circadian | cis-acting regulatory element involved in circadian control |
| CnSBP10 | GT1-motif | light-responsiveness element |
| CnSBP10 | GT1-motif | light-responsiveness element |
| CnSBP10 | GT1-motif | light-responsiveness element |
| CnSBP10 | I-box | light-responsiveness element |
| CnSBP10 | TCT-motif | light-responsiveness element |
| CnSBP10 | GATA-motif | light-responsiveness element |
| CnSBP10 | GATA-motif | light-responsiveness element |
| CnSBP10 | G-box | light-responsiveness element |
| CnSBP10 | ABRE | auxin-responsive element |
| CnSBP10 | ABRE | auxin-responsive element |
| CnSBP10 | ARE | cis-acting regulatory element essential for the anaerobic induction |
| CnSBP10 | ARE | cis-acting regulatory element essential for the anaerobic induction |
| CnSBP10 | CGTCA-motif | cis-acting regulatory element involved in the MeJA-responsiveness |
| CnSBP10 | HD-Zip | element involved in differentiation of the palisade mesophyll cells |
| CnSBP10 | MBS | MYB binding site involved in drought-inducibility |
| CnSBP10 | O2-site | cis-acting regulatory element involved in zein metabolism regulation |
| CnSBP10 | circadian | cis-acting regulatory element involved in circadian control |
| CnSBP11 | GT1-motif | light-responsiveness element |
| CnSBP11 | GT1-motif | light-responsiveness element |
| CnSBP11 | GT1-motif | light-responsiveness element |
| CnSBP11 | GT1-motif | light-responsiveness element |
| CnSBP11 | Sp1 | light-responsiveness element |
| CnSBP11 | I-box | light-responsiveness element |
| CnSBP11 | TCT-motif | light-responsiveness element |
| CnSBP11 | G-Box | light-responsiveness element |
| CnSBP11 | ABRE | auxin-responsive element |
| CnSBP11 | ABRE | auxin-responsive element |
| CnSBP11 | ARE | cis-acting regulatory element essential for the anaerobic induction |
| CnSBP11 | CGTCA-motif | cis-acting regulatory element involved in the MeJA-responsiveness |
| CnSBP11 | TGACG-motif | cis-acting regulatory element involved in the MeJA-responsiveness |
| CnSBP11 | HD-Zip | element involved in differentiation of the palisade mesophyll cells |
| CnSBP11 | O2-site | cis-acting regulatory element involved in zein metabolism regulation |
| CnSBP11 | circadian | cis-acting regulatory element involved in circadian control |
| CnSBP12 | 3-AF1 binding site | light-responsiveness element |
| CnSBP12 | Gap-box | light-responsiveness element |
| CnSBP12 | GA-motif | light-responsiveness element |
| CnSBP12 | I-box | light-responsiveness element |
| CnSBP12 | ARE | cis-acting regulatory element essential for the anaerobic induction |
| CnSBP12 | ARE | cis-acting regulatory element essential for the anaerobic induction |
| CnSBP12 | CGTCA-motif | cis-acting regulatory element involved in the MeJA-responsiveness |
| CnSBP12 | TGACG-motif | cis-acting regulatory element involved in the MeJA-responsiveness |
| CnSBP12 | GCN4_motif | cis-regulatory element involved in endosperm expression |
| CnSBP12 | GCN4_motif | cis-regulatory element involved in endosperm expression |
| CnSBP13 | GT1-motif | light-responsiveness element |
| CnSBP13 | GT1-motif | light-responsiveness element |
| CnSBP13 | GT1-motif | light-responsiveness element |
| CnSBP13 | I-box | light-responsiveness element |
| CnSBP13 | chs-CMA1a | light-responsiveness element |
| CnSBP13 | chs-CMA1a | light-responsiveness element |
| CnSBP13 | Box 4 | light-responsiveness element |
| CnSBP13 | Box 4 | light-responsiveness element |
| CnSBP13 | ARE | cis-acting regulatory element essential for the anaerobic induction |
| CnSBP13 | ARE | cis-acting regulatory element essential for the anaerobic induction |
| CnSBP13 | ARE | cis-acting regulatory element essential for the anaerobic induction |
| CnSBP13 | ARE | cis-acting regulatory element essential for the anaerobic induction |
| CnSBP13 | CGTCA-motif | cis-acting regulatory element involved in the MeJA-responsiveness |
| CnSBP13 | TGACG-motif | cis-acting regulatory element involved in the MeJA-responsiveness |
| CnSBP13 | LTR | cis-acting element involved in low-temperature responsiveness |
| CnSBP13 | MBS | MYB binding site involved in drought-inducibility |
| CnSBP13 | MBS | MYB binding site involved in drought-inducibility |
| CnSBP13 | P-box | gibberellin-responsive element |
| CnSBP14 | GT1-motif | light-responsiveness element |
| CnSBP14 | GT1-motif | light-responsiveness element |
| CnSBP14 | GA-motif | light-responsiveness element |
| CnSBP14 | TCT-motif | light-responsiveness element |
| CnSBP14 | TCT-motif | light-responsiveness element |
| CnSBP14 | ARE | cis-acting regulatory element essential for the anaerobic induction |
| CnSBP14 | AuxRR-core | cis-acting regulatory element involved in auxin responsiveness |
| CnSBP14 | CAT-box | cis-acting regulatory element related to meristem expression |
| CnSBP14 | HD-Zip | element involved in differentiation of the palisade mesophyll cells |
| CnSBP14 | MBS | MYB binding site involved in drought-inducibility |
| CnSBP14 | O2-site | cis-acting regulatory element involved in zein metabolism regulation |
| CnSBP14 | TGA-element | auxin-responsive element |
| CnSBP14 | TGACG-motif | cis-acting regulatory element involved in the MeJA-responsiveness |
| CnSBP14 | CGTCA-motif | cis-acting regulatory element involved in the MeJA-responsiveness |
| CnSBP15 | GT1-motif | light-responsiveness element |
| CnSBP15 | GT1-motif | light-responsiveness element |
| CnSBP15 | GT1-motif | light-responsiveness element |
| CnSBP15 | G-box | light-responsiveness element |
| CnSBP15 | G-box | light-responsiveness element |
| CnSBP15 | G-box | light-responsiveness element |
| CnSBP15 | G-box | light-responsiveness element |
| CnSBP15 | ABRE | auxin-responsive element |
| CnSBP15 | ABRE | auxin-responsive element |
| CnSBP15 | ABRE | auxin-responsive element |
| CnSBP15 | ABRE | auxin-responsive element |
| CnSBP15 | ABRE | auxin-responsive element |
| CnSBP15 | ABRE | auxin-responsive element |
| CnSBP15 | ABRE | auxin-responsive element |
| CnSBP15 | ARE | cis-acting regulatory element essential for the anaerobic induction |
| CnSBP15 | CGTCA-motif | cis-acting regulatory element involved in the MeJA-responsiveness |
| CnSBP15 | TGACG-motif | cis-acting regulatory element involved in the MeJA-responsiveness |
| CnSBP15 | O2-site | cis-acting regulatory element involved in zein metabolism regulation |
| CnSBP15 | TC-rich repeats | cis-acting element involved in defense and stress responsiveness |
| CnSBP15 | TCA-element | cis-acting element involved in salicylic acid responsiveness |
| CnSBP15 | TCA-element | cis-acting element involved in salicylic acid responsiveness |
| CnSBP15 | TCA-element | cis-acting element involved in salicylic acid responsiveness |
| CnSBP15 | TGA-element | auxin-responsive element |
| CnSBP16 | GT1-motif | light-responsiveness element |
| CnSBP16 | GT1-motif | light-responsiveness element |
| CnSBP16 | GT1-motif | light-responsiveness element |
| CnSBP16 | L-box | light-responsiveness element |
| CnSBP16 | Sp1 | light-responsiveness element |
| CnSBP16 | G-box | light-responsiveness element |
| CnSBP16 | G-box | light-responsiveness element |
| CnSBP16 | chs-CMA1a | light-responsiveness element |
| CnSBP16 | ABRE | auxin-responsive element |
| CnSBP16 | ABRE | auxin-responsive element |
| CnSBP16 | ABRE | auxin-responsive element |
| CnSBP16 | ARE | cis-acting regulatory element essential for the anaerobic induction |
| CnSBP16 | ARE | cis-acting regulatory element essential for the anaerobic induction |
| CnSBP16 | ARE | cis-acting regulatory element essential for the anaerobic induction |
| CnSBP16 | ARE | cis-acting regulatory element essential for the anaerobic induction |
| CnSBP16 | ARE | cis-acting regulatory element essential for the anaerobic induction |
| CnSBP16 | ARE | cis-acting regulatory element essential for the anaerobic induction |
| CnSBP16 | CAT-box | cis-acting regulatory element related to meristem expression |
| CnSBP16 | CGTCA-motif | cis-acting regulatory element involved in the MeJA-responsiveness |
| CnSBP16 | CGTCA-motif | cis-acting regulatory element involved in the MeJA-responsiveness |
| CnSBP16 | CGTCA-motif | cis-acting regulatory element involved in the MeJA-responsiveness |
| CnSBP16 | CGTCA-motif | cis-acting regulatory element involved in the MeJA-responsiveness |
| CnSBP16 | TGACG-motif | cis-acting regulatory element involved in the MeJA-responsiveness |
| CnSBP16 | TGACG-motif | cis-acting regulatory element involved in the MeJA-responsiveness |
| CnSBP16 | TGACG-motif | cis-acting regulatory element involved in the MeJA-responsiveness |
| CnSBP16 | TGACG-motif | cis-acting regulatory element involved in the MeJA-responsiveness |
| CnSBP16 | GCN4-motif | cis-regulatory element involved in endosperm expression |
| CnSBP16 | TCA-element | cis-acting element involved in salicylic acid responsiveness |
| CnSBP16 | HD-Zip | element involved in differentiation of the palisade mesophyll cells |
| CnSBP16 | circadian | cis-acting regulatory element involved in circadian control |
| CnSBP17 | GT1-motif | light-responsiveness element |
| CnSBP17 | LAMP-element | light-responsiveness element |
| CnSBP17 | TCT-motif | light-responsiveness element |
| CnSBP17 | TCT-motif | light-responsiveness element |
| CnSBP17 | TCT-motif | light-responsiveness element |
| CnSBP17 | GATA-motif | light-responsiveness element |
| CnSBP17 | ABRE | auxin-responsive element |
| CnSBP17 | ABRE | auxin-responsive element |
| CnSBP17 | ARE | cis-acting regulatory element essential for the anaerobic induction |
| CnSBP17 | ARE | cis-acting regulatory element essential for the anaerobic induction |
| CnSBP17 | ARE | cis-acting regulatory element essential for the anaerobic induction |
| CnSBP17 | AuxRR-core | cis-acting regulatory element involved in auxin responsiveness |
| CnSBP17 | CGTCA-motif | cis-acting regulatory element involved in the MeJA-responsiveness |
| CnSBP17 | CGTCA-motif | cis-acting regulatory element involved in the MeJA-responsiveness |
| CnSBP17 | TGACG-motif | cis-acting regulatory element involved in the MeJA-responsiveness |
| CnSBP17 | TGACG-motif | cis-acting regulatory element involved in the MeJA-responsiveness |
| CnSBP17 | circadian | cis-acting regulatory element involved in circadian control |
| CnSBP17 | TATC-box | cis-acting element involved in gibberellin-responsiveness |
| CnSBP17 | TC-rich repeats | cis-acting element involved in defense and stress responsiveness |
| CnSBP17 | TCA-element | cis-acting element involved in salicylic acid responsiveness |
| CnSBP17 | MBS | MYB binding site involved in drought-inducibility |
| CnSBP18 | AE-box | light-responsiveness element |
| CnSBP18 | AE-box | light-responsiveness element |
| CnSBP18 | G-Box | light-responsiveness element |
| CnSBP18 | LAMP-element | light-responsiveness element |
| CnSBP18 | LAMP-element | light-responsiveness element |
| CnSBP18 | ABRE | auxin-responsive element |
| CnSBP18 | ABRE | auxin-responsive element |
| CnSBP18 | ARE | cis-acting regulatory element essential for the anaerobic induction |
| CnSBP18 | CAT-box | cis-acting regulatory element related to meristem expression |
| CnSBP18 | MBS | MYB binding site involved in drought-inducibility |
| CnSBP18 | MBS | MYB binding site involved in drought-inducibility |
| CnSBP19 | GT1-motif | light-responsiveness element |
| CnSBP19 | TCT-motif | light-responsiveness element |
| CnSBP19 | G-box | light-responsiveness element |
| CnSBP19 | AE-box | light-responsiveness element |
| CnSBP19 | ABRE | auxin-responsive element |
| CnSBP19 | ABRE | auxin-responsive element |
| CnSBP19 | ABRE | auxin-responsive element |
| CnSBP19 | ARE | cis-acting regulatory element essential for the anaerobic induction |
| CnSBP19 | ARE | cis-acting regulatory element essential for the anaerobic induction |
| CnSBP19 | ARE | cis-acting regulatory element essential for the anaerobic induction |
| CnSBP19 | CAT-box | cis-acting regulatory element related to meristem expression |
| CnSBP19 | CGTCA-motif | cis-acting regulatory element involved in the MeJA-responsiveness |
| CnSBP19 | CGTCA-motif | cis-acting regulatory element involved in the MeJA-responsiveness |
| CnSBP19 | TGACG-motif | cis-acting regulatory element involved in the MeJA-responsiveness |
| CnSBP19 | TGACG-motif | cis-acting regulatory element involved in the MeJA-responsiveness |
| CnSBP19 | TCA-element | cis-acting element involved in salicylic acid responsiveness |
| CnSBP19 | TCA-element | cis-acting element involved in salicylic acid responsiveness |
| CnSBP19 | TGA-element | auxin-responsive element |
| CnSBP20 | GT1-motif | light-responsiveness element |
| CnSBP20 | GT1-motif | light-responsiveness element |
| CnSBP20 | TCCC-motif | light-responsiveness element |
| CnSBP20 | G-box | light-responsiveness element |
| CnSBP20 | AE-box | light-responsiveness element |
| CnSBP20 | ATC-motif | light-responsiveness element |
| CnSBP20 | ABRE | auxin-responsive element |
| CnSBP20 | ABRE | auxin-responsive element |
| CnSBP20 | ABRE | auxin-responsive element |
| CnSBP20 | AuxRR-core | cis-acting regulatory element involved in auxin responsiveness |
| CnSBP20 | LTR | cis-acting element involved in low-temperature responsiveness |
| CnSBP20 | MBS | MYB binding site involved in drought-inducibility |
| CnSBP20 | TCA-element | cis-acting element involved in salicylic acid responsiveness |
| CnSBP21 | 3-AF1 binding site | light-responsiveness element |
| CnSBP21 | 3-AF1 binding site | light-responsiveness element |
| CnSBP21 | GT1-motif | light-responsiveness element |
| CnSBP21 | GT1-motif | light-responsiveness element |
| CnSBP21 | Gap-box | light-responsiveness element |
| CnSBP21 | TCT-motif | light-responsiveness element |
| CnSBP21 | TCT-motif | light-responsiveness element |
| CnSBP21 | G-box | light-responsiveness element |
| CnSBP21 | G-box | light-responsiveness element |
| CnSBP21 | G-box | light-responsiveness element |
| CnSBP21 | G-box | light-responsiveness element |
| CnSBP21 | ABRE | auxin-responsive element |
| CnSBP21 | ABRE | auxin-responsive element |
| CnSBP21 | ABRE | auxin-responsive element |
| CnSBP21 | ABRE | auxin-responsive element |
| CnSBP21 | ABRE | auxin-responsive element |
| CnSBP21 | ABRE | auxin-responsive element |
| CnSBP21 | ARE | cis-acting regulatory element essential for the anaerobic induction |
| CnSBP21 | CAT-box | cis-acting regulatory element related to meristem expression |
| CnSBP21 | CGTCA-motif | cis-acting regulatory element involved in the MeJA-responsiveness |
| CnSBP21 | TGACG-motif | cis-acting regulatory element involved in the MeJA-responsiveness |
| CnSBP21 | HD-Zip | element involved in differentiation of the palisade mesophyll cells |
| CnSBP21 | HD-Zip | element involved in differentiation of the palisade mesophyll cells |
| CnSBP21 | LTR | cis-acting element involved in low-temperature responsiveness |
| CnSBP21 | LTR | cis-acting element involved in low-temperature responsiveness |
| CnSBP21 | MBS | MYB binding site involved in drought-inducibility |
| CnSBP21 | O2-site | cis-acting regulatory element involved in zein metabolism regulation |
| CnSBP21 | GCN4-motif | cis-regulatory element involved in endosperm expression |
| CnSBP21 | TC-rich repeats | cis-acting element involved in defense and stress responsiveness |
| CnSBP21 | TC-rich repeats | cis-acting element involved in defense and stress responsiveness |
| CnSBP21 | TCA-element | cis-acting element involved in salicylic acid responsiveness |
